# The PD-1/PD-L1 Pathway: A Perspective on Comparative Immuno-Oncology

**DOI:** 10.3390/ani12192661

**Published:** 2022-10-04

**Authors:** Sandra Schöniger, Bharat Jasani

**Affiliations:** Discovery Life Sciences Biomarker Services GmbH, Germaniastrasse 7, 34119 Kassel, Germany

**Keywords:** programmed death protein 1, PD-1, programmed death protein ligand 1, PD-L1, comparative pathology, one health one medicine concept, immuno-oncology, cancer

## Abstract

**Simple Summary:**

The programmed cell death protein 1/programmed death-ligand 1 (PD-1/PD-L1) pathway inhibits the function of activated immune cells. This mediates immune tolerance and prevents immune-mediated tissue destruction. The malfunction of this pathway is involved in the pathogenesis of chronic infections, autoimmunity, and cancer. The PD-1/PD-L1 pathway is an excellent example of the research benefits of comparative pathology and attests to the importance of the “one health one medicine” concept. Pioneering research was mainly focused on the examination of cells and tissues of human and mouse origin. It mainly revealed that PD-L1-positive tumor cells can paralyze PD-1-bearing immune cells, which prevents immunological destruction of cancer cells. This led to a major breakthrough in cancer treatment, i.e., the use of antibodies that block the interaction of these molecules and restore anti-cancer immune defense (immune checkpoint therapy). Further studies provided more detailed information on the tissue-specific context and fine-tuning of this pathway. The most recent research has extended the investigations to the examination of several animal species with the aim of improving disease diagnostics and treatment for certain animal diseases, in particular cancer, which is a major cause of disease and death in companion animals.

**Abstract:**

The programmed cell death protein 1/programmed death-ligand 1 (PD-1/PD-L1) pathway mainly attracted attention in immuno-oncology, leading to the development of immune checkpoint therapy. It has, however, much broader importance for tissue physiology and pathology. It mediates basic processes of immune tolerance and tissue homeostasis. In addition, it is involved in the pathogenesis of chronic infectious diseases, autoimmunity, and cancer. It is also an important paradigm for comparative pathology as well as the “one health one medicine” concept. The aim of this review is to provide an overview of novel research into the diverse facets of the PD-1/PD-L1 pathway and to give insights into its fine-tuning homeostatic role in a tissue-specific context. This review details early translational research from the discovery phase based on mice as animal models for understanding pathophysiological aspects in human tissues to more recent research extending the investigations to several animal species. The latter has the twofold goal of comparing this pathway between humans and different animal species and translating diagnostic tools and treatment options established for the use in human beings to animals and vice versa.

## 1. Introduction

The programmed cell death protein 1/programmed death-ligand 1 (PD-1/PD-L1) pathway from its discovery phase to the present constitutes an excellent example of the “one health one medicine” concept.

It is based on the fact that humans and domestic animals share the same environment and may develop similar diseases, and that there is a mutual benefit of exchanging scientific information on medical conditions as well as available treatment options between physicians and veterinarians [1]. It provides a working hypothesis to identify animal models for certain human diseases and to explore whether treatment options available for humans may be suitable for animals as well [1]. 

The overall research on the PD-1/PD-L1 pathway has revealed an increasing complexity of this pathway that has implications for developing basic knowledge into physiological mechanisms to understand pathological alterations and provide insights into reasons for immuno-oncological treatment success and failure. 

The authors consider this review as a valuable contribution to the existing literature on the PD-1/PD-L1 pathway due to its focus on comparative pathology and the “one health one medicine” aspect with particular emphasis on immuno-oncology. 

## 2. The Discovery Phase of the PD-1/PD-L1 Pathway

### 2.1. The Discovery of PD-1

The PD-1 receptor was discovered in 1992 during studies on the apoptotic elimination of self-reactive immature T cells within the thymus conducted by Tasuku Honjo and colleagues at Kyoto University, Japan, giving rise to its name “programmed cell death protein 1” (PD-1) [2]. Subsequently, genes specifically activated during apoptosis were investigated in cell lines in which apoptosis could be induced artificially by exposure to ionomycin/phorbol 12-myristate 13-acetate (PMA) or growth factor (i.e., interleukin 3) deprivation [2]. The identified gene (PD-1 gene) was found to represent a novel member of the immunoglobulin gene superfamily [2]. It encodes a predicted protein sequence of 288 amino acids with two hydrophobic regions located at the amino (N) terminus (constituting a signal peptide) and the middle region, respectively [2]. Cleavage of the N-terminal signal peptide leads to the mature PD-1 protein composed of 268 amino acids, which encompasses a 147 amino acid long extracellular domain with four possible N-glycosylation sites, a 27 amino acid long transmembrane domain, and an intracellular domain with 94 amino acids [2]. To reveal the tissue distribution of PD-1, Northern hybridization was performed on messenger ribonucleic acids (mRNAs) extracted from different mouse tissues (thymus, spleen, brain, kidney, liver, lungs, and heart) [2]. Unequivocal PD-1 mRNA expression was detected in the thymus, and a probable weak expression was detected in the spleen and lungs, whereas the remaining tissues were regarded as negative [2]. 

The human homolog of the PD-1 gene (PDCD1) was identified in 1994 by the screening of a human T cell complementary deoxyribonucleic acid (cDNA) library with a mouse PD-1 cDNA probe [3]. The deduced human PD-1 protein amino acid sequence shared 60% sequence homology with the mouse PD-1 protein [3].

Furthermore, PD-1 was identified as a 50–55 kDa transmembrane protein that is expressed on murine lymphocytes [4]. Whereas unstimulated T- and B-lymphocytes show low PD-1 expression levels, marked PD-1 upregulation is detected after stimulation mediated by T cell receptors (TCRs) and B cell receptors [4]. PD-1-positive T cells included not only CD4-positive (CD4+) or CD8-positive (CD8+) T cells, but also CD4 and CD8 double-negative (CD4-/CD8-) thymocytes [4]. The designation “PD-1” was retained, although observed results contradicted the original perception that PD-1 represents an apoptosis-specific protein [2,4]. Instead, the new evidence clearly showed that PD-1 expression mainly occurred as a secondary event to lymphocyte activation [4]. The ionomycin/PMA-mediated PD-1 induction may be explained by treatment-associated activation of protein kinase C and elevation of intracellular calcium that also occurs after TCR stimulation [4]. It was hypothesized that PD-1 modulates effector functions of lymphocytes and plays a role during T cell development in the thymus [4]. 

The latter assumption was further studied by Nishimura et al. [5]. In the murine thymus, PD-1 protein was detected on about 34% of CD4-/CD8- thymocytes that included thymocytes of the αβ and γδ TCR lineages as well as αβ TCR natural killer (NK) cells [5]. In CD4-/CD8- αβ TCR thymocytes, the increase in PD-1 paralleled the expression of the β-chain of the TCR that leads to stimulation through the TCR. This was also mimicked by injection of mice with anti-CD3 antibody, which markedly increased the expression of PD-1 on CD25 and CD44 double-negative thymocytes of the αβ TCR lineage [5]. 

The identification of the ligand of PD-1 [6,7] also highlighted its role as an inhibitory costimulatory molecule on effector T cells [6,7]. The inhibitory function correlates with the presence of an immunoreceptor tyrosine-based inhibitory motif (ITIM) in its cytoplasmic region [8].

### 2.2. The Discovery of PD-L1 and PD-L2

The first report on the ligand for PD-1 was published by Dong et al. in 1999 [6]. Initially, this molecule was named B7 homolog 1 (B7-H1) since it was revealed as a further member of the B7 receptor family [6]. In an independent study, Freemann et al. [7] also identified the ligands of human and murine PD-1 that were named murine and human programmed death ligand 1 (PD-L1) [7]. Later, it turned out that B7-H1 and PD-L1 were identical molecules. In this review, only the designation PD-L1 is used.

PD-L1 consists of 290 amino acids and belongs to the B7 receptor family [6,7]. The human and murine PD-L1 proteins share 70% sequence homology [7]. They display a similar structural organization, i.e., an immunoglobulin variable and immunoglobulin constant domain containing an extracellular region, a hydrophobic transmembrane domain, and a short charged intracellular domain [6,7]. Other members of the B7 family are B7-1 (CD80) and B7-2 (CD86) as well as inducible co-stimulator (ICOS) [6,7]. 

In humans and mice, PD-L1 mRNA is expressed by antigen-presenting cells (APCs) and lymphocytes, and a strong upregulation occurs after interferon γ (IFNγ) treatment [7]. The expression of B7-1 (CD80) and B7-2 (CD86) molecules on APCs is also stimulated by IFNγ [7]. Similarly, PD-L1 protein is detected in only scant amounts in unstimulated B and T cells, whereas about 16% of CD14-positive (CD14+) monocytes display a constitutive expression of PD-L1 [6]. A strong PD-L1 upregulation is induced by phytohemagglutinin in CD3-positive T cells (about 30% positive cells) and by IFNγ and lipopolysaccharide (LPS) in CD14+ monocytes (about 90% positive cells), whereas LPS increased the percentage of PD-L1-positive B cells to a lesser degree (about 6% positive cells) [6]. 

In general, PD-L1 mRNA is detected in human [6,7] and murine tissues [7], with high levels occurring in the heart, skeletal muscle, placenta, and lungs and low levels in the kidney, spleen, and liver [6,7]. In the thymus, Dong et al. [6] detected low PD-L1 mRNA expression whilst Freemann et al. [7] found high PD-L1 mRNA expression. Most importantly, when comparing murine T cells from wild-type (wt) mice and PD-1-deficient mice, it was shown that PD-L1 binding to PD-1 on lymphocytes inhibits TCR-mediated lymphocyte proliferation [7]. Similarly, the addition of PD-L1 to activated human CD4+ T cells inhibited their proliferation and cytokine secretion [7]. Notably, it was further demonstrated that the degree of inhibition can be modulated by the strength of the TCR activation together with the intensity of the co-stimulation exerted by the binding of CD28 with CD80 and/or CD86 [7]. 

Subsequently, programmed cell death ligand 2 (PD-L2) was discovered as a further ligand of PD-1 (synonym: B7-DC) [9,10]. Human and murine PD-L2 mRNA shows a similar tissue distribution to PD-L1 mRNA; in particular, high levels are present in the placenta [9]. 

## 3. The Physiological Expression Patterns of PD-L1 and PD-1

### 3.1. Co-Stimulation and Inhibition

**Figure 1:**
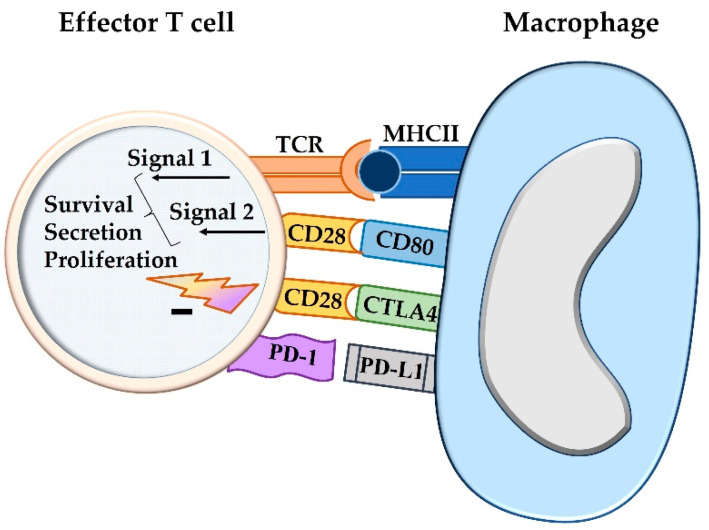
The activation state of a T cell, i.e., cytokine production, proliferation, and overall survival, is not only influenced by co-stimulation, but also by concurrent inhibitory signals. The two-signal hypothesis refers to the antigen presentation by major histocompatibility complex I or II molecules of an antigen-presenting cell that causes antigen priming of the T cell receptor (signal 1). For activation of a T cell, co-stimulation through ligation of CD80 or CD86 with CD28 is required (signal 2). The degree of the activation, however, can be further modulated by additional stimulatory or inhibitory signals. The latter are mediated by CTLA4, which competes with CD80/CD86 for CD28 binding, and by the interaction between PD-L1 and PD-1, both acting as immune checkpoint molecules. TCR = T cell receptor; MHCII = major histocompatibility complex II; CTLA4 = cytotoxic T-lymphocyte-associated protein 4; PD-1 = programmed cell death protein 1; PD-L1 = programmed death-ligand 1.

PD-1, PD-L1, PD-L2, and cytotoxic T-lymphocyte-associated protein 4 (CTLA-4) have all been found to act as immune checkpoint molecules [11]. In recent years, it has been revealed that the intensity of an immune reaction can be modulated by the involvement of additional costimulatory and inhibitory signals [11]. During the priming of naïve lymphocytes in the lymphoid organ, the inhibitory ligand CTLA-4 competes with CD28 for the binding of CD80/CD86 on the antigen-presenting cell, and this attenuates the intensity of the induced immune response [11,12]. In contrast, an inhibitory impact on activated effector T cells is mediated by the interaction between PD-L1 or PD-L2 on antigen-presenting cells and PD-1 on effector lymphocytes [11,12] (Figure 1). 

This was also confirmed by in vitro studies based on a comparison of T cells isolated from PD-1 knockout (KO) mice and wt mice [13]. In co-culture with PD-L1-positive cells, activated T cells from PD-1 KO mice show a higher proliferative rate and higher cytokine secretion than those obtained from wt mice [13], and the impaired proliferation of wt T cells was abolished by the addition of a PD-L1-blocking antibody [13]. 

### 3.2. Intracellular Signal Transduction Pathways

The cytoplasmic tail of PD-1 contains an immunoreceptor tyrosine-based inhibition motif (ITIM) and an immunoreceptor tyrosine-based switch motif (ITSM) [14]. After binding between PD-1 and PD-L1 or PD-L2, the tyrosine residues in these two domains become phosphorylated. This in turn activates Src homology region 2 (SH2) domain-containing tyrosine phosphatase-2 (SHP-2) and the homologous SH2 domain-containing tyrosine phosphatase-1 (SHP-1). The sequel is the dephosphorylation of CD28 and to a lesser degree TCR and other costimulatory molecules with inhibition of signal transduction and thus a reduction in cell proliferation, cytokine secretion, and survival [14,15,16,17]. This is the classical way of interaction referred to as “trans” interaction [14]. 

### 3.3. Cell Populations Expressing PD-1, PD-L1, and PD-L2

**Figure 2:**
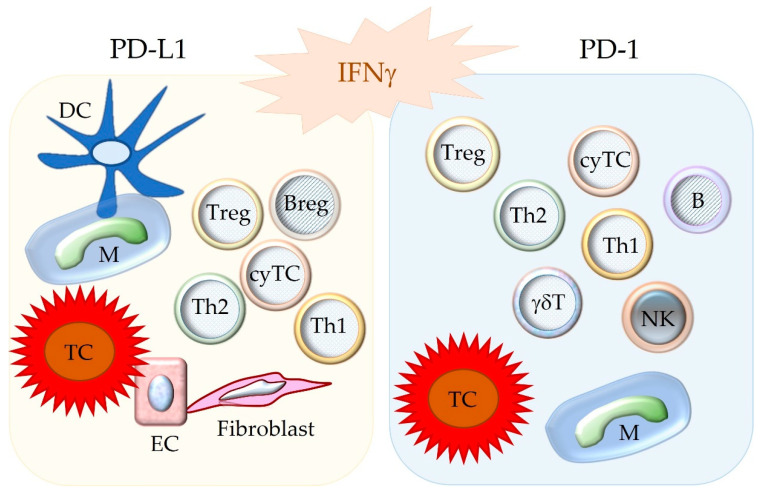
Depicted are cell populations with possible expression of the immune checkpoint molecules PD-L1 and PD-1. Notably, these molecules are upregulated by IFNγ. The binding between PD-L1 and PD-1 inhibits the functions of the PD-1-bearing target cell. DC = dendritic cell; M = macrophage; TC = tumor cell; EC = endothelial cell; Treg = regulatory T cell; Breg = regulatory B cell; B = B cell, Th1 = T helper 1 cell; Th2 = T helper 2 cell; cyTC = cytotoxic T cell; NK = natural killer cell; γδT = γδ T cell.

Subsequent to the pioneering studies described above, it has been revealed that PD-1 is expressed in many different immune cell populations. These include different subtypes of activated and exhausted T cells including cytotoxic T cells (cyTCs), B cells, natural killer cells, monocytes, macrophages, dendritic cells, and myeloid precursor cells [14,16,18,19,20]. Further, PD-1 can be detected on cancer cells of some tumor entities as well [14,21,22]. Its ligand PD-L1 is found on APCs, T cells, and other immune cells [14,18,23]. It is also expressed by non-hematopoietic cells including vascular endothelial cells and islet cells of the endocrine pancreas [14,18] as well as cancer cells of numerous tumor types [12,24]. In comparison, PD-L2 shows a more restricted distribution to mainly APCs [10,14,18], although it can also be found on neoplastic cells of some tumors [12,21] (Figure 2).

Throughout pregnancy, in the human placenta PD-L1 and PD-L2 are expressed in different cell populations of the fetal and maternal site including syncytiotrophoblasts, cytotrophoblasts, and decidual stromal cells [25], whereas PD-1 is upregulated on decidual T cells [25]. Thus, these immune checkpoint molecules contribute—together with other immune regulatory mechanisms—to the active immunotolerance of pregnancy and protect the fetus against immunological rejection [25]. 

PD-1 and PD-L1 can be either expressed in different cell populations or simultaneously on the same cells such as antigen-presenting cells, activated T cells, or cancer cells [14,21,26].

### 3.4. Functions of PD-L1 and PD-1 in Tissues 

Physiological functions of the PD-1/PD-L1 pathway include induction of central and peripheral tolerance, maintenance of immune privilege status, and the downregulation of an immune response.

PD-1 modulates the signaling thresholds for positive selection of thymocytes, whereas reduced expression of PD-1 or PD-L1 correlates with a higher percentage of positively selected thymocytes [27].

The presence of PD-L1 on vascular endothelial cells restricts the tissue access of T cells [13]. In addition, the PD-1/PD-L1 pathway prevents the activation of autoreactive T cells [27,28]. The interaction of PD-L1 expressed by APCs and PD-1 on naïve T cells induces and sustains Treg formation and function [29]. This is caused by inhibition of the Akt kinase/mammalian target of rapamycin (mTOR) signaling cascade and stimulation of expression of the transcription factor forkhead box protein P3 (Foxp3) [29].

The PD-L1/PD-1 pathway contributes—together with other immunomodulatory factors—to the tolerance of foreign antigens in immune privilege sites, i.e., the anterior chamber of the eye, testis, brain, central nervous system, and placenta [30]. 

During an acute immune response, effector T cells rapidly upregulate PD-1. This attenuates or terminates T cell activation and prevents excessive tissue damage [28]. 

## 4. Dysfunction of the PD-1/PD-L1 Pathway

### 4.1. Loss of Inhibition: Autoimmune Disease 

Dysregulation of the PD-1/PD-L1 pathway predisposes to autoimmune disease, as is demonstrated by in vitro and in vivo studies on animal models. Disease manifestation in PD-1 knockout mice is influenced by their genetic background. For example, those on a B6 background show lupus-like illness with glomerulonephritis and arthritis [5], whereas those on a BALB/c background develop dilative cardiomyopathy [31,32] and those on a “non-obese diabetic” (NOD) background develop diabetes mellitus type I [33]. Moreover, PD-L1 and PD-L2 have distinct roles in the regulation of autoimmunity and disease susceptibility. For example, PD-L1 KO mice are more prone to experimental autoimmune encephalomyelitis (EAE) than PD-L2 KO mice or control animals [28]. The application of anti-PD-1 or anti-PD-L1 antibodies to mice with autoimmune diseases (EAE, diabetes mellites, enteritis) accelerated the disease condition [28]. 

In human beings, the manifestation of autoimmune diseases has been associated with *PDCD1* gene polymorphisms [28,34,35,36]; examples are rheumatoid arthritis [34], systemic lupus erythematosus [35], and multiple sclerosis [36]. High levels of soluble PD-1 molecules, which are detected in rheumatoid arthritis, can block the inhibitory action of PD-L1 and sustain inflammatory reactions [28]. In systemic lupus erythematosus, decreased PD-1 expression is detected in active disease manifestation [28]. In multiple sclerosis, intralesional T cells lack PD-1 and thus cannot be inhibited by PD-L1 expression in the brain tissue [28]. In addition, autoimmune reactions can occur as a side effect of immune checkpoint therapy [28]. 

### 4.2. T Cell Exhaustion Caused by Chronic Immune Stimulation 

In acute infections, short-lived effector cells (SLECs) and memory precursor effector cells (MPECs) develop [37]. Antigen exposure transiently induces low-level PD-1 expression on effector T cells, which ceases after antigen clearance [37]. This is followed by a contraction phase that is characterized by cytokine-deprivation-induced apoptosis of SLECs and the formation of memory CD8+ T cells by MPECs [37,38]. 

A long duration of antigenic stimulation, for example in the context of chronic infections and neoplastic disease, facilitates T cell exhaustion, which is associated with chronic upregulation of PD-1 [18,39]. 

PD-1 blockade can lead to a reinvigoration of exhausted T cells [18]. 

Recent research has revealed that exhausted cyTCs are composed of interconnected subpopulations that are within different stages of development and express different levels of PD-1 [37]. Stem-like cyTCs with low PD-1 expression and self-renewing properties develop into effector cyTCs with low PD-1 expression that further differentiate into terminally dysfunctional cyTCs with high PD-1 levels [37]. The latter cells often bear additional co-inhibitory receptors, such as CTLA-4, T cell immunoglobulin mucin-3 (Tim-3), T cell immunoreceptor with immunoglobulin and immunoreceptor tyrosine-based inhibitory motif domains (TIGIT), and lymphocyte-activation gene 3 (LAG-3) [37]. In addition, they acquire epigenetic changes that impair or even completely prevent the full restoration of their effector functions after immune checkpoint inhibition [37]. Those terminally dysfunctional cyTCs, which constantly express high PD-1 levels, have an increased susceptibility to cell death since PD-1-associated signaling pathways involve the upregulation of pro-apoptotic molecules [14,18,37]. Thus, it has been recognized that the success of immune checkpoint inhibition is influenced by the relative frequency of the individual subsets of exhausted T cells in the tumor microenvironment (TME), whereas only those T cell subsets prior to the stage of terminal dysfunction are amenable to complete functional reinvigoration [37]. 

#### 4.2.1. Chronic Infectious Diseases

Chronic viral, bacterial, or parasitic infections are commonly associated with the aforementioned T cell exhaustion [39]. Several of these chronic infections constitute major global health problems since they cause severely debilitating diseases with high mortality rates due to final organ failure [39]. Examples are hepatitis B due to infection with hepatitis B virus, acquired human immunodeficiency syndrome caused by human immunodeficiency virus, tuberculosis induced by infection with Mycobacterium tuberculosis, and infection with Plasmodium falciparum as the causative agent of malaria [39]. 

Notably, not only chronic antigenic stimulation, but also organ-specific features modulate the time course of an infection. For example, the liver sinusoidal endothelial cells and Kupffer cells express high levels of PD-L1, which binds to PD-1 of T cells and inhibits their effector functions [13]. On the one hand, this prevents an immune response against dietary antigens or commensal bacteria transported to the liver via the portal vein; on the other hand, it may impair the elimination of infectious agents [13]. 

Similar immuno-suppressive effects are also often observed in tumor tissue; i.e., the exhaustion of T cells is accompanied by PD-L1 expression on tumor cells [12]. 

#### 4.2.2. Neoplastic Disease

PD-L1 mRNA was found to be expressed in several murine tumor cell lines, including those of lymphoid tumors, mastocytoma, hepatoma, neuroblastoma, and breast cancer [9]. Subsequently, PD-L1 was observed on neoplastic cells of all examined murine myeloma cell lines [40]. Considering the PD-L1-mediated inhibition of effector T cells, it was hypothesized that PD-L1-positive tumors downregulate anti-tumor cytotoxic defenses of immune cells, particularly cyTCs [40]. This was confirmed by several experimental approaches [40]. PD-L1-positive tumor cells were less susceptible to cytolysis by effector T cells in vitro and showed enhanced tumorigenesis and invasiveness in wt mice [40]. The application of an anti-PD-L1 antibody reversed these effects [40]. After subcutaneous injection of highly immunogenic PD-L1-positive myeloma cells, tumors developed in wt mice, but not in PD-1 KO mice [40]. In contrast, however, the subcutaneous injection of poorly immunogenic melanoma cells caused enhanced tumor cell growth only in PD-1 transgenic mice, while no differences in tumor volumes were detected between wt mice and PD-1 knockout mice [41]. These findings reveal that in most tumors PD-L1 expression is evoked by an anti-tumor Th1 immune response resulting in the release of IFNγ [41]. Since tumors with higher antigenicity elicit a more severe inflammatory response, they display higher PD-L1 expression levels and show increased suppression of anti-tumor immune responses in comparison to poorly immunogenic tumors [41].

Notably, hematogenous dissemination of tumor cells was more severe in wt mice than in PD-1 KO mice and was reversed by the intraperitoneal injection of PD-1-blocking antibodies in wt mice [41]. This finding can be explained by higher percentages of activated CD4+ and CD8+ T cells in PD-1 KO mice together with their increased recruitment to tumor sites as well as their higher proliferation and cytokine secretion rates [41].

## 5. The Complexity of the PD-L1/PD-1 Pathway in Cancer Biology 

### 5.1. Mechanisms of PD-L1 Expression in Cancer Cells

The expression of PD-L1 on cancer cells can be adaptive or develop as a sequel to mutations [12,22,24]. The latter type is named constitutive oncogenic expression and evokes a PD-L1 staining of all tumor cells [22,24]. This can be caused by different types of mutations including amplification of chromosome 9, which contains the locus of PD-L1, PD-L2, and the interferon receptor adapter Janus kinase 2 (JAK2), as well as epidermal growth factor receptor (EGFR) mutations, phosphatase and tensin homolog (PTEN) deletions, phosphatidylinositol-3-kinase/protein kinase B (PI3K/PKB) mutations, and c-myelocytomatosis oncogene product (c-myc) overexpression [24]. Adaptive induced PD-L1 expression is the sequel of the presence of T cells in the TME that are activated by tumor antigens and secrete high IFNγ levels [24]. Thus, it is commonly observed in tumors with marked T cell infiltration, i.e., so-called “hot tumors” [42]. Additional proinflammatory cytokines as well as hypoxia can contribute to the induced expression of PD-L1 on tumor cells [22]. Within the tumor, this evokes a patchy staining pattern [24]. PD-L1-positive tumor cells are mostly observed in areas with T cell infiltration as well as at the invasive tumor margin [24]. Since immune cells are equipped with IFNγ receptors, upon exposure to IFNγ, PD-L1 is also upregulated on lymphocytes and macrophages in the TME [24]. Constitutive oncogenic and adaptive induced PD-L1 expression can also occur in combination [24]. 

**Figure 3:**
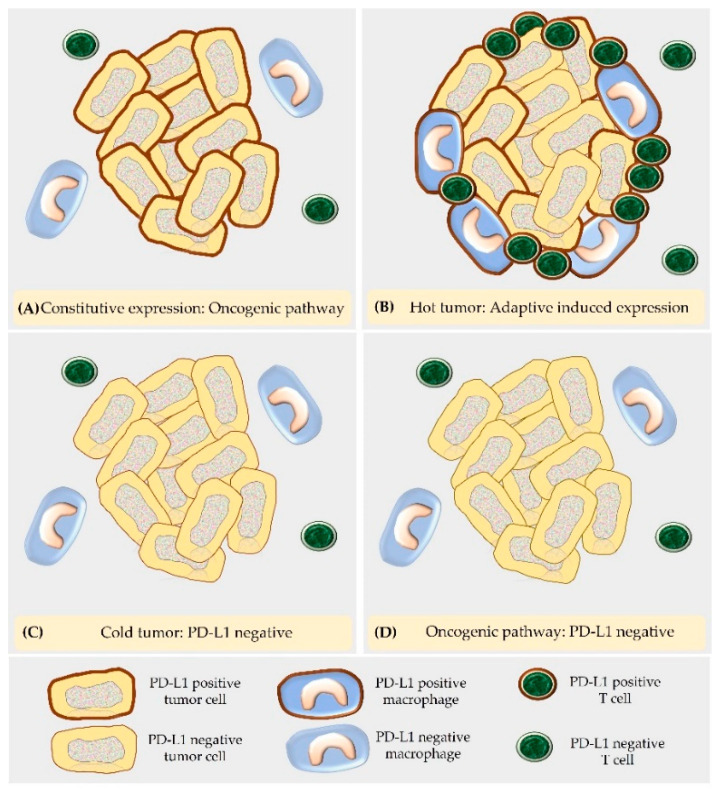
According to the underlying molecular mechanism, PD-L1 expression in tumor cells can be classified in four different categories, i.e., PD-L1-positive by the constitutive oncogenic pathway (**A**), PD-L1-positive by the adaptive induced PD-L1 expression in a “hot tumor” (**B**), PD-L1-negative in a “cold tumor” (**C**) and PD-L1-negative by the oncogenic pathway (**D**).

**Figure 4:**
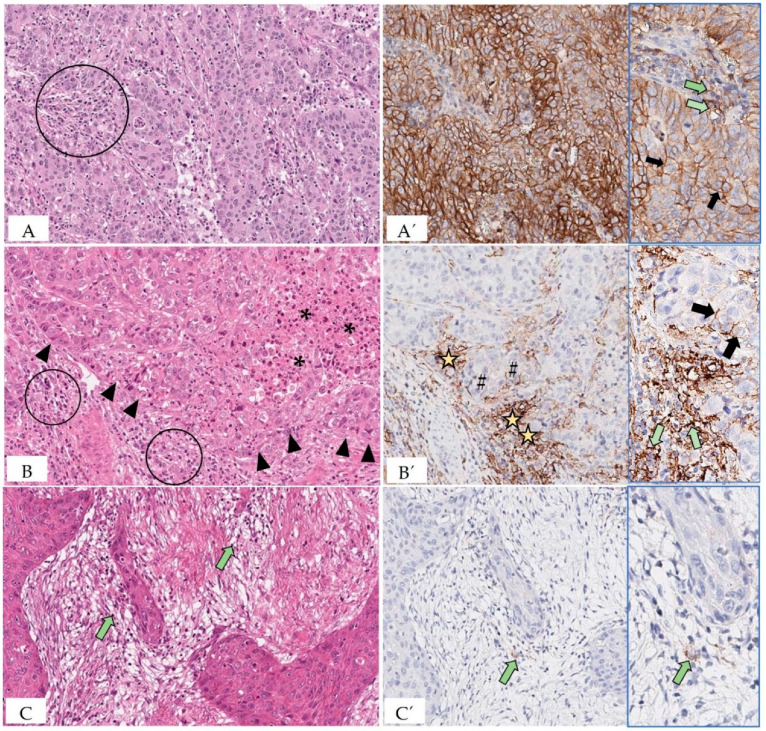
Depicted are representative areas of sections of human carcinomas that are stained with hematoxylin–eosin (**A**–**C**) and immunolabeled for PD-L1 (**A’**–**C’**). Immunostaining was performed with PD-L1 IHC 22C3 pharmDx for Autostainer Link 48 and DAB as chromogen. **A** (carcinoma of the gastro-esophageal junction): The scarce tumor stroma contains mild to moderate numbers of tumor-infiltrating lymphocytes (TILs; circle). 20×. **A’**: All tumor cells show linear partial to complete moderate to strong membranous staining consistent with constitutive PD-L1 expression through the oncogenic pathway. 20×. Inset: Shown in greater detail is the membrane staining of tumor cells (black arrows). In addition, a few TILs have membranous to cytoplasmic PD-L1 expression (green arrows). **B** (esophageal carcinoma): The tumor stroma is infiltrated with moderate TIL numbers (circles). The interface between stroma and tumor cell nests is demarcated by arrowheads. An area of necrosis is marked by asterisks. 20×. **B’**: PD-L1-positive immune cells accumulate at the interface between tumor cell nests and stroma (stars). The PD-L1 staining of tumor cells is restricted to those that are located at the periphery of the tumor cell nests in immediate proximity to the PD-L1-positive immune cells (hash signs). This staining pattern is consistent with adaptive induced PD-L1 expression. 20×. Inset: In higher magnification are depicted aggregates of PD-L1-positive immune cells that show a strong membranous to cytoplasmic staining (green arrows). The partial to complete membranous staining (usually of a weaker intensity compared to the adjacent immune cells) of the cancer cells is marked by black arrows. 40×. **C** (carcinoma of the gastro-esophageal junction): The tumor stroma is infiltrated by only a few TILs (green arrows). 20× **C’**: Tumor cells are PD-L1 immunonegative, whereas very few immune cells are PD-L1-positive (green arrow). 20×. Inset: The PD-L1-positive immune cell (green arrow) is depicted at higher magnification.

PD-L1-negative staining of tumor cells can be classified into two categories, i.e., PD-L1-negative tumors lacking an immune cell infiltrate (“cold tumors”) and tumors with constitutive PD-L1-negative immunostaining. Within “cold tumors,” neoplastic cells have the property to acquire PD-L1 immunostaining in response to any event that increases their immunogenicity and evokes an immune response [24]. Constitutive negative PD-L1 staining is the result of mutations, e.g., those that prevent signaling through the IFNγ receptor [24]. Such a mutation can also develop as a sequel to immune checkpoint inhibition [24] (Figure 3 and Figure 4). 

The knowledge of the mechanisms underlying these staining patterns has particular relevance for the diagnostic interpretation of PD-L1 immunostaining. Thus, the response to immune checkpoint inhibition requires not only PD-L1 labeling of tumor cells, but also the presence of immune cells. As consequence, neoplasms with the adaptive PD-L1 expression will generally show a favorable response to immune checkpoint therapy [24].

### 5.2. “Hot Tumors” and “Cold Tumors”

Tumors with numerous immune cells in the TME are named “hot tumors”, whereas those with no or only sparse immune cell infiltrates represent “cold tumors” [42]. The marked immune cell infiltration of “hot tumors” is explained by their high immunogenicity [42]. This is associated with chronic exposure of tumor antigens to immune cells and leads to exhaustion of infiltrating immune cells that upregulate PD-1 [18,42]. 

The high immunogenicity of “hot tumors” can be caused by different molecular mechanisms. For example, it may be associated with mismatch repair deficiency (dMMR) and/or high microsatellite instability (MSI-H) [43,44,45] or a major histocompatibility complex class II (MHCII) signature [46,47]. Thus, dMMR/MSI-H [43,44,45] or MHII signature [46,47] serve as independent and robust biomarkers for predicting a favorable immune response to immune checkpoint inhibition. 

Whereas all nucleated cells contain major histocompatibility complex class I (MHC I) molecules, MHC II molecules are only present on APCs, but may also be expressed by cancer cells [46,47]. They can be induced or upregulated by IFNγ exposure [46]. Antigens presented through MHC II molecules activate CD4+ T helper 1 cells, whereas those bound to MHC I stimulate cyTCs [46].

Within the TME, cancer cell-mediated MHC II restricted antigen presentation provides an APC-independent mechanism to activate CD4+ T helper 1 cells [46]. Further, cancer cells and APCs may present different antigens through MHC II molecules [46]. Cancer cells can also provide co-stimulation, e.g., by CD80 and intercellular adhesion molecule 1 (ICAM-1) that bind to CD28 and lymphocyte function-associated antigen 1 (LFA-1) on T cells, respectively [46].

Notably, the activation of effector and memory cyTCs by antigens bound to MHC I molecules is promoted by CD4+ T helper 1 cells [46]. Most cyTCs are CD8+; however, a subgroup of CD4+ cyTCs exists, which can also directly destroy tumor cells by cytolytic action [48]. CD4+ T cells are also required for the induction of humoral immune responses against tumor antigens [14].

### 5.3. Exosomal and Serum-Derived PD-1 and PD-L1 Molecules

It has been shown that cancer cells of different tumor entities can shed PD-L1-containing exosomes in the circulation [49]. These can inhibit cellular immune responses within the TME, the draining lymph node, and the circulation [49]. Notably, in triple-negative breast cancer, the exosomal secretion of PD-1 by TILs has been reported [50]. Exosomal PD-1 can induce internalization of PD-L1 present on tumor cells through clathrin-mediated endocytosis [50]. Further, it can capture PD-L1 molecules in the circulation, and thus it may help to preserve anti-cancer cellular immune defenses [50]. Serum PD-1 and PD-L1 molecules can be derived from exosomes, or they can represent the soluble form of these molecules [51]. Soluble PD-1 or PD-L1 molecules may be formed by cleavage of their extracellular domains or from alternative splicing of the pre-mRNA [51]. The relevance of these molecules as predictive and prognostic biomarkers in different cancers is currently under investigation [51]. 

### 5.4. The Concept of “Trans” and “Cis” Interaction 

**Figure 5:**
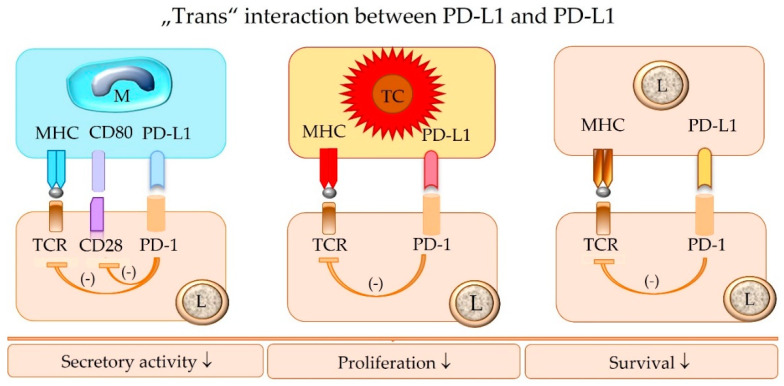
The “trans” interaction of PD-1 of an effector lymphocyte (L) with PD-L1 expressed by a macrophage (M), tumor cell (TC), or another lymphocyte (L) inhibits the functional activity of the PD-1-bearing target lymphocyte (L), including its survival, proliferation, and secretory activity. TCR = T cell receptor; MHC = major histocompatibility complex class I or II molecules; PD-1 = programmed cell death protein 1; PD-L1 = programmed death-ligand 1.

The terminology “trans” interaction is used to describe the binding between PD-L1 and PD-1 expressed on two different cells [14,26]. The “trans” interaction between PD-L1 of an APC or a tumor cell and PD-1 of an effector T cell mediates the canonical inhibitor signaling pathway [14,26] (Figure 5). 

**Figure 6:**
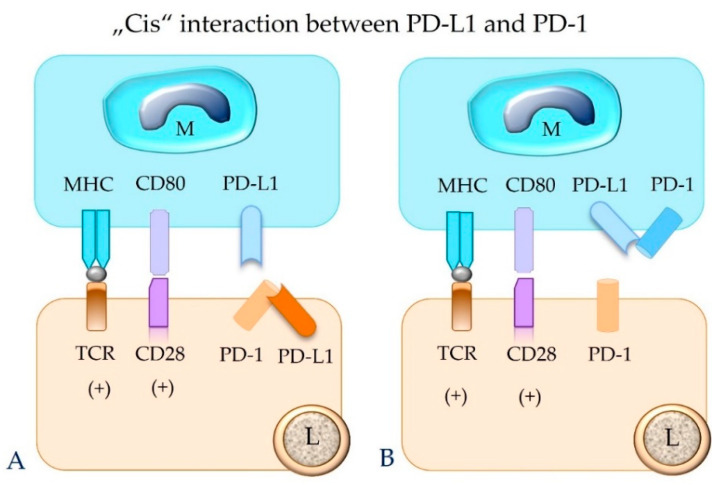
The binding between PD-L1 and PD-1 molecules that are expressed on the same cell (“cis” ligation) will hinder their “trans” interaction. This can result in failure of functional inhibition of the effector T cell. Depicted is “cis” interaction between PD-1 and PD-L1 in a lymphocyte (**A**) and a macrophage (**B**). M = macrophage; L = lymphocyte; TCR = T cell receptor; MHC = major histocompatibility complex class I or II molecule; PD-1 = programmed cell death protein 1; PD-L1 = programmed death-ligand 1.

In contrast, the same immune or tumor cell can also express both PD-1 and PD-L1 on its surface, and an interaction between these molecules can occur, which is designated “cis” binding [14,22,26]. “Cis” binding between these two molecules competes with their “trans” interaction, and thus it can alleviate T cell inhibition [14,22,26] (Figure 6). 

The net result of the interaction between PD-L1 and PD-1 is likely further influenced by their relative numbers on the same cell and different cells of one or several cell populations. There is evidence that this may have implications for the response to immune checkpoint therapy as well [14,22,52]. 

**Figure 7:**
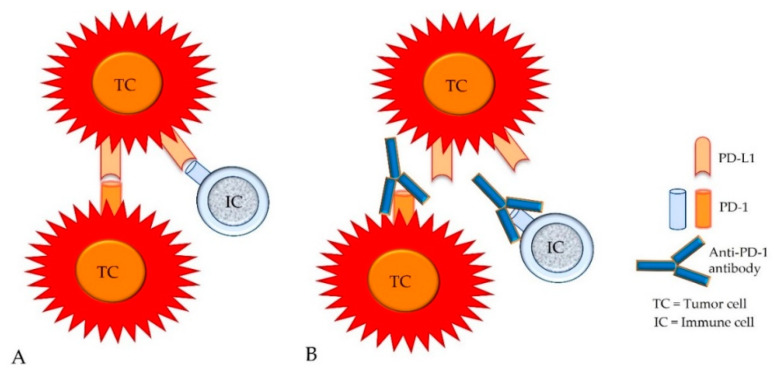
In addition to the expression of PD-L1, the intrinsic expression of PD-1 has been detected on variable numbers of tumor cells from different cancer types. Therefore, PD-L1 molecules of tumor cells (TCs) can bind not only to PD-1 molecules of immune cells (ICs), but also to PD-1 molecules of tumor cells (**A**). Both types of cellular interactions can be blocked by anti-PD-1 antibodies (**B**).

The simultaneous expression of intrinsic PD-1 and intrinsic or adaptive PD-L1 has also been reported on subsets of tumor cells, whereas these molecules can be detected on different tumor cells or on the same tumor cell [21,22,52,53,54]. Their “trans” interaction appears to evoke tumor-type-specific effects [21,22,52,53,54]. In melanomas, this can activate mTOR signaling and the subsequent phosphorylation of ribosomal protein S6 (RPS6) which results in tumor cell proliferation [21,22,53]. Similarly, also in hepatocellular carcinoma and carcinoma of the urinary bladder, the activation of PD-1 on tumor cells by PD-L1 expressed on other tumor cells or on immune cells may stimulate tumor cell proliferation [21,22,54]. The opposite effect, however, may be evoked in some cases of non-small-cell lung cancer and colon cancer, in which the ligation of tumor cell-associated PD-1 by PD-L1 was found to inhibit neoplastic cell proliferation [21,22,52]. Variable numbers of PD-1-positive tumor cells (mRNA and protein expression) can be detected in additional cancer types such as carcinoids, bladder cancer, urothelial carcinoma, and testicular carcinoma [21,22] (Figure 7).

### 5.5. The Concept of “Forward” and “Reverse” Signaling

PD-L1 of an immune cell can act as a ligand that induces signaling in the PD-1-bearing target cell (forward signaling) as well as a receptor that receives signaling through its ligation with PD-1 (reverse signaling) [14,55]. For example, PD-L1 of a lymphocyte can bind PD-1 expressed on an activated T cell or a macrophage [14]. In a forward signaling mode, T cell tolerance or conversion of an M1 macrophage to an M2 macrophage is mediated [14,55]. Macrophages with the M1 phenotype exhibit anti-tumorigenic action since they have the capabilities to lyse and phagocytize cancer cells [56]. In contrast, macrophages with the M2 functional differentiation facilitate tumor growth and progression. They stimulate angiogenesis and neovascularization as well as stromal remodeling and activation. Further, they release immunosuppressive cytokines [56]. 

**Figure 8:**
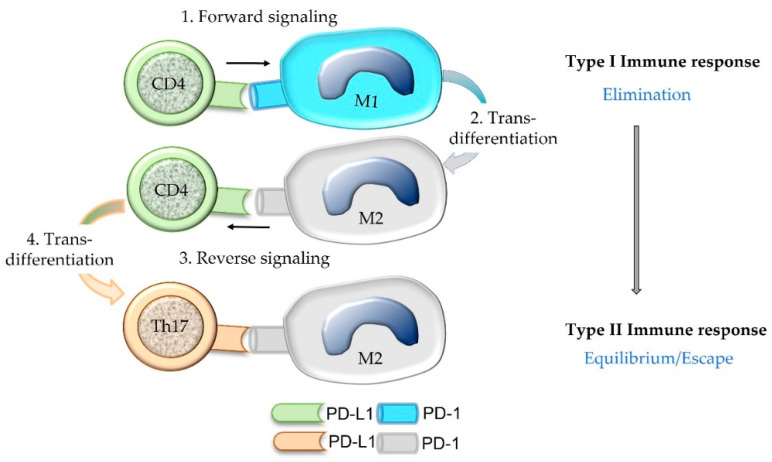
Binding of the ligand PD-L1 expressed on a CD4+ Th1 cell to its receptor PD-1 present on an M1 macrophage can induce M2 trans-differentiation of the PD-1-bearing target cell (forward signaling). Subsequently, the PD-1-bearing M2 macrophage may evoke trans-differentiation of the CD4+ Th1 cell into a Th17 cell by a process named reverse signaling. Within the tumor microenvironment, such a trans-differentiation of immune cells may contribute to the shifting of an anti-cancer type I immune response to a type II immune response that is associated with an equilibrium between cancer and immune cells or cancer immune escape.

Reverse signaling in CD4+ T cells may trigger Th17 polarization and inhibits T helper cell differentiation, whereas in CD8+ T cells it inhibits cytotoxic functions [14,55] (Figure 8). Thus, tolerogenic effects are not only induced by PD-L1-positive tumor cells and PD-L1-positive APCs, but also by PD-L1-positive T cells [55]. These data provide the scientific rationale for the combined evaluation of PD-L1-positive immune and cancer cells constituting the combined positive score (CPS) for the diagnostic determination of eligibility for immune checkpoint therapy.

There is evidence for the existence of reverse signaling through PD-L1 expressed on tumor cells of selected cancer types leading to tumor initiation and progression, metabolic reprogramming, and resistance to therapy [22]. 

### 5.6. Additional Receptors for PD-L1 and PD-L2

**Figure 9:**
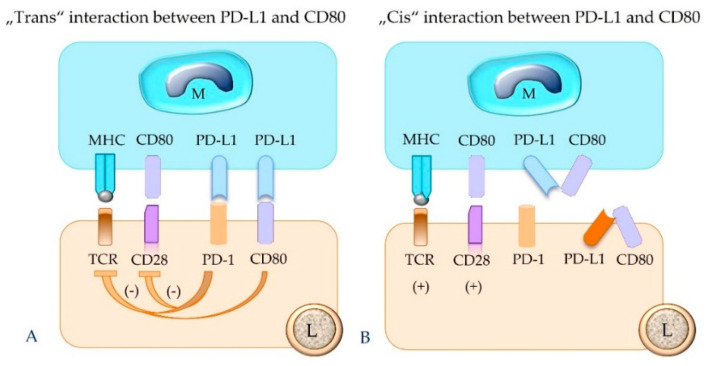
PD-L1 expressed by a macrophage (M, as depicted), tumor cell, or regulatory T cell can also bind to CD80 of a lymphocyte (L). This results in inhibition of the functional activity of the PD-1-bearing target cell (**A**). The “cis” interaction of PD-L1 and CD80 molecules on the same cell, e.g., a macrophage or lymphocyte, prevents their binding in “trans” and thus counteracts their inhibitory function (**B**). TCR = T cell receptor; MHC = major histocompatibility complex class I or II molecule; PD-1 = programmed cell death protein 1; PD-L1 = programmed death-ligand 1.

It has been shown that PD-L1 also binds to CD80 and PD-L2 to repulsive guidance molecule B [14,18]. Human CD80 shows differences in affinity to its binding partners PD-L1, CD28, and CTLA-4; i.e., it has the highest affinity for CTLA-4 and the lowest for CD28 [57]. Notably, CD80 is present not only on APCs, but also on activated T cells [57] and tumor cells [22,58]. These molecules can either be expressed by different cell populations or be displayed on the same cell. Therefore, interactions can also occur through “trans” and “cis” binding [14] (Figure 9).

### 5.7. Evaluation of PD-L1 Status of Tumor and Immune Cells 

To determine the eligibility of a patient for immune checkpoint therapy, immunostaining for PD-L1 may be performed. Available assays differ in regard to the primary antibodies, staining platforms, and cut-off values for immunostained tumor cells and/or immune cells (SP142; 22C3; 28-8) [59]. In tumor cells, positive staining is characterized as partial to complete membrane staining of any intensity readily discernible at ×20 objective lens setting. In contrast, immune cells (lymphocytes and macrophages) are defined as positive if they display a membranous and/or cytoplasmic reaction [60,61,62] (Figure 4). 

### 5.8. PD-1 Expression on Myeloid Precursor Cells 

The role of myeloid-specific PD-1 was demonstrated by Strauss et al. [19], who created a mouse model with conditional ablation of the PD-1 gene in myeloid cells. Emergent myelopoiesis within the TME leads to the accumulation of granulocyte and macrophage progenitors (GMPs) that are PD-1-positive and differentiate into myeloid-derived suppressor cells (MDSCs) [19]. Mice with myeloid-specific ablation of PD-1 lacked GMPs and MDSCs, had increased numbers of systemic effector myeloid cells and T effector memory cells, and showed inhibition of tumor growth [19]. In addition, compared to PD-1-proficient myeloid cells, PD-1-deficient myeloid cells displayed markedly elevated cholesterol. The latter is essential for the development of M1 macrophages and dendritic cells and stimulates antigen presentation [19]. These findings suggest that immune checkpoint therapy also acts through metabolic reprogramming and redifferentiation of myeloid cells [19].

## 6. Beyond Humans and Mice

Initially, mice were used as animal models to decipher the functions of PD-1, PD-L1, and PD-L2 in human tissues [2,4,5]. Subsequently, the insights obtained from studies on murine and human cells and tissues have been applied to examine the functional role of these molecules in different animal species. For this, it has been necessary to develop antibodies detecting PD-1 and PD-L1 or PD-L2 in different animal species. These include anti-canine PD-1 [63], anti-canine PD-L1 [63,64], anti-bovine PD-L1 [65], and anti-bovine PD-1 [66]. Anti-bovine PD-L1 antibody shows cross-reactivity with porcine PD-L1 [67] and canine PD-L1 [68]. In addition, cross-reactive anti-human PD-1, PD-L1, or PD-L2 antibodies were used in canine [69], equine [70], and feline tissues [71].

Interestingly, transgenic pigs have been produced with the expression of human PD-L1 (hPD-L1) mRNA and protein in different organs and peripheral blood mononuclear cells (PBMCs) [72]. About 12–16% of PBMCs in these mice were found to be hPD-L1-positive; these included T cells (CD3+, CD4+, CD8+), B cells, and monocytes [72]. After exposure to concanavalin A and interleukin 2 (IL-2), the percentage of hPD-L1-expressing PBMCs was significantly increased (69–80%) [72]. 

Due to the popularity of dogs as companion animals and the frequent occurrence of cancer in older dogs [73], numerous studies have been focused on the expression of immune checkpoint molecules in canine tumors.

### 6.1. Chronic Infectious Diseases

In different animal species, chronic disease is also associated with exhaustion of immune cells. 

In cattle, the expression of PD-1 and/or PD-L1 on immune cells is elevated during the late stage of bovine leukemia virus infection [74] as well as chronic infections with Mycoplasma bovis [65] and Anaplasma marginale [75]. In general, higher numbers of PD-L1- or PD-1-positive immune cells were associated with lower IFNγ production, and the addition of PD-L1- or PD-1-blocking antibodies raised IFNγ levels [65,74,75]. There was a positive correlation between a high pathogen load and increased numbers of PD-L1- or PD-1-positive immune cells [74,75]. 

Splenic tissue of dogs with chronic leishmaniosis contained significantly higher numbers of PD-1-, PD-L1-, and PD-L2-positive immune cells than that of uninfected control dogs [69]. Labeling of immune cells was cytoplasmic and/or membranous [69]. 

In pigs, an increase in PD-L1 immunostaining has been described in tissue samples of pigs with chronic diseases, i.e., infection with Mycoplasma hyopneumoniae, Lawsonia intracellularis, and porcine circovirus 2, compared to uninfected tissue from a control pig [67]. 

The comparison of blood samples from cats with and without chronic feline immunodeficiency virus infection revealed that infected cats had significantly higher numbers of PD-1- and PD-L1-positive lymphocytes [76].

### 6.2. Neoplastic Disease

The examination of 20 equine penile squamous cell carcinomas revealed PD-L1 mRNA and protein in three cases and one case, respectively, whereas PD-1 expression was not detected [70].

Serum levels of soluble PD-1 and PD-L1 were significantly higher in cats with human epidermal growth factor receptor 2 positive (HER2+) and triple-negative (negative for estrogen and progesterone receptors and HER2) normal-like mammary carcinomas than in cats with other molecular subtypes of mammary carcinoma and those without mammary neoplasm [71]. Further, HER2+ tumors showed a higher PD-L1 expression in cancer cells and tumor-infiltrating lymphocytes (TILs) than triple-negative normal-like mammary carcinomas [71]. The PD-L1 immunostaining in TILs was reported as cytoplasmic or membranous, whereas the labeling in tumor cells was described as localized to cytoplasmic or nuclear membranes [71].

Most canine cancer cell lines, including those of melanomas, round cell tumors, and different types of carcinomas and sarcomas, and cultures of canine macrophages displayed a constitutive or IFNγ-induced PD-L1 expression [68,77]. Further, nearly all cell lines with a constitutive PD-L1 expression—except a lymphoma cell line—showed increased PD-L1 expression after exposure to IFNγ [77]. The treatment of cell lines of canine melanoma, osteosarcoma, transitional cell carcinoma, and histiocytic sarcoma with a ligand of Toll-like receptor 3 elevated PD-L1 levels on tumor cells [77]. The examination of PD-1 and PD-L1 in canine urothelial cancer cell lines confirmed the presence of both molecules, whereas PD-1 levels were consistently higher than PD-L1 levels [78]. In addition, PD-L1 expression was also detected in single-cell suspensions of canine tumors (angiosarcoma, carcinomas) [68]. PD-L1 immunopositive tumor cells were observed in different canine neoplasms (melanomas, sarcomas, carcinomas, round cell tumors) [68,79], and described staining patterns were cytoplasmic and membranous [79]. Moreover, the presence of PD-1-positive TILs was confirmed as well [79]. RNA scope revealed PD-L1 and PD-1 mRNA in tumor cells of canine diffuse large B cell lymphoma as well as PD-1 mRNA in TILs [80].

To also provide potential immune checkpoint therapy to dogs, Maekawa et al. [81] produced a chimeric anti-canine PD-L1 monoclonal antibody. The in vitro application of this antibody resulted in higher cytokine production and increased proliferation of stimulated canine blood mononuclear cells compared to those without antibody exposure [81]. In a pilot clinical trial involving seven dogs with malignant oral melanoma and two dogs with undifferentiated sarcoma, the application of this antibody resulted in tumor regression in one dog with oral melanoma and in one dog with undifferentiated sarcoma [81]. 

## 7. Discussion

### 7.1. Immuno-Oncology

Immune checkpoint therapy uses antibodies that bind to PD-1 and/or PD-L1. These hinder the interaction of both molecules and reverse the inhibition of the PD-1-bearing target cell. Within tumor tissue, these antibodies prevent the binding between PD-L1 of tumor cells and PD-1 of lymphocytes and thereby restore the anti-cancer immune responses [12], whereby the cytolysis of tumor cells is mainly mediated by CD8+ cyTCs [12]. To determine the eligibility of a patient for immune checkpoint therapy, the presence of PD-L1-positive tumor cells and/or immune cells is analyzed [24] or the presence of dMMR or MSI-H is determined [43,44,82]. It was noted, however, that not all tumors fulfilling these criteria show long-term remission after immune checkpoint therapy [24].

From the discovery phase of the PD-L1/PD-1 pathway until now, the knowledge on this pathway has continuously expanded. In particular, it has been revealed that the effects of the interaction between PD-L1 and PD-1 are markedly influenced not only by cell populations bearing these molecules, but also by their relative frequencies as well as their distribution within the tissue. 

The complex interactions of tumor cells and different types of immune cells are best visualized and examined by multiplex immunohistochemistry or immunofluorescence. For interpretation of the results, it is to be considered that any particular analysis is only a “snapshot of time”: Immune cells show a high plasticity, and their differentiation is influenced by changes in the TME [83], which can be influenced by hypoxia or certain treatments such as chemotherapy or radiation. Moreover, the interaction of immune cells and/or tumor cells is only possible if they are located within a certain distance from each other [84].

### 7.2. Comparative Pathology

During the discovery phase studies of the PD-1/PD-L1 pathway, most examinations were performed on human and mouse cell lines and tissue with the aim to reveal aspects that are relevant to human medicine [2,4,5]. It is to be noted, however, that some differences in the expression patterns of PD-L1 and PD-1 exist between humans and mice [57].

More recently, the PD-L1/PD-1 pathway has also been examined in farm animals and companion animals to determine whether these molecules play a role in diseases of these animal species as well. 

In farm animals, these studies have mainly centered on chronic infectious diseases due to their economic relevance [65,66,67,74,75], whereas in companion animals, investigations have focused on canine cancer [64,77,79,81]. Notably, the estimated annual cancer rate in dogs is higher than that in human beings. In the USA, about 500 per 100.000 people are diagnosed with cancer, whereas approximately 5300 per 100.000 dogs receive this diagnosis [73]. 

The results obtained so far on the PD-1/PD-L1 pathway in animals provide important insights into comparative pathology and will likely help to find novel treatment options for chronic infections and neoplastic diseases in different animal species. Widespread investigations across different animal species, however, are limited by intra- and interspecies differences in disease susceptibility and the limited availability of cross-reactive anti-PD-1 or anti-PD-L1 antibodies.

In human medicine, standardized evaluation schemes for the interpretation of immunohistochemical results exist that provide guidance for treatment decisions. These differ amongst a plethora of available diagnostic antibodies and assays, associated different staining platforms, and the respective tumor entity or indication [59]. For a direct comparison of studies on human and animal tissues, the use of identical evaluation schemes or protocols would be of major advantage.

Interestingly, the knowledge about the PD-1/PD-L1 pathway has also been explored in the generation of transgenic pigs as donors for human organ transplants [72]. These pigs carry the hPD-L1 gene, and hPD-L1 on tissues and organs of transgenic pigs reacts only with human PD-1, but not with porcine PD-1, and its expression levels can be modulated by human cytokines [72]. 

## 8. Conclusions

This review summarizes the multiple facets of the PD-1/PD-L1 pathway and emphasizes its central role for comparative pathology and the “one health one medicine” concept. The mutual exchange of knowledge and experience about the regulation of this pathway in humans and different animal species will not only increase our insight into this pathway but also improve diagnostics and treatment options in human and veterinary medicine. The basic principles for this type of interdisciplinary research are already established. A comparison of research data, however, is only possible if standardized guidelines for their acquisition and interpretation exist. Therefore, the establishment of such guidelines is regarded as the next step in obtaining data on the PD-1/PD-L1 pathway that can be compared across humans and different animal species.
